# Supplementary material for: Knowledge, attitudes, and practices towards childhood tuberculosis among healthcare workers at two primary health facilities in Lusaka, Zambia
Source: PLoS One. 2024 Mar 11;19(3):e0287876. doi: 10.1371/journal.pone.0287876 (PMC10927107; doi:10.1371/journal.pone.0287876)
Supplement: S1 File — (DOCX) [file pone.0287876.s002.docx]

| Participant ID |
| --- |

**Appendix 8: KAP survey questionnaire for health workers**

**KAP survey questionnaire on knowledge, attitudes and practices of healthcare workers towards childhood TB**

Date: ___ /___ / ___(DD/MM/YYYY)

Health facility ____________________

Instructions to data collector: Please circle appropriate response

# General and demographic questions

1. How old are you?
   1. Under 30
   2. 31–40
   3. 41–50
   4. Over 50

1. What is your gender?
   1. Male
   2. Female

1. What is your profession?
   1. Nurse
   2. Clinical officer
   3. Medical Doctor

1. In which department do you work?
   1. Outpatient department
   2. ART
   3. In-patient department
   4. MCH
   5. TB
   6. Nutrition ward

1. For how long have you been working at this health facility?
   1. Less than 1 year
   2. Between 1- 3 years
   3. Between 5-3 years
   4. Between 10-5 years
   5. More than 10 years
2. Have you ever had a training on TB?
   1. Yes
   2. No > Q#9

1. What were you trained on? (Circle all that apply.)
   1. Adult TB
   2. Childhood TB
   3. 3 “Is”
   4. TB preventive therapy
   5. Infection control
   6. Intensified case finding
   7. TB/HIV management

1. How long ago were you trained?
   1. Less than 3 months
   2. Less than 6 months
   3. Less than 1 year
   4. More than 1 year
   5. More than 3 years

| TB knowledge |
| --- |
| Epidemiology and transmission |

1. Zambia is a high burden TB country (Circle one.)
   1. Yes
   2. No
   3. Not sure

1. How is TB spread? (Circle all that apply)
   1. Coughing
   2. Sneezing
   3. Singing
   4. Laughing
   5. Skin contact

1. TB can affect the following body parts? (Circle all that apply)
   1. Lungs
   2. Larynx
   3. Heart
   4. Spine
   5. Meninges
   6. Lymph nodes
   7. Abdomen
   8. Pleura

1. Extra-pulmonary TB is common in children? (Circle one.)
   1. Yes
   2. No
   3. Not sure

1. Which of the following is a risk factor for childhood TB **infection**? (Please circle all that are mentioned.)
   1. Not being vaccinated with BCG
   2. HIV
   3. Malnutrition
   4. Being less than 5 years old
   5. Living in Zambia
   6. Being a contact to a TB case

# Diagnosis of TB

1. What are the symptoms of TB in children? (Please circle all that apply.)
   1. Cough
   2. Low appetite
   3. Tiredness/reduced playfulness
   4. Weight loss
   5. Fever
   6. Chest pain
   7. Shortness of breath 8. Do not know

9. Other:

1. Which of the following is a risk factor for childhood TB **disease**? (Please circle all that apply.)
   1. Not being vaccinated with BCG
   2. HIV
   3. Malnutrition
   4. Being less than 5 years old
   5. Living in Zambia
   6. Being a contact to a TB case

1. What is the first line TB diagnostic test in Zambia (Circle only one answer )
   1. Chest x-ray
   2. Gene Xpert
   3. Gene Xpert ultra
   4. Smear microscopy 5. Do not know

6. Other:

1. How many samples are needed for Gene Xpert (Circle only one answer )
   1. One spot sample
   2. One early morning sample
   3. Two samples: one spot and one in the early morning
   4. Two spot samples
   5. Three samples: One spot sample, an early morning sample and another spot sample
2. Which of the following diagnostic tests can be used to diagnose drug resistant TB (Circle all that are mentioned)
   1. Chest x-ray
   2. Gene Xpert
   3. Gene Xpert ultra
   4. Smear microscopy 10. Do not know

11. Other:

# Treatment of TB

1. How long is the treatment of uncomplicated pulmonary TB in children? (Please circle one answer.)
   1. 1 month
   2. 6 months
   3. 12 months
   4. Not sure

1. Which of the following is correct treatment for children with uncomplicated pulmonary TB in children? (Please circle one answer.)
   1. 2HERZ/4HR
   2. 2HRZ/4HR
   3. 2HERZ/10HR
   4. 2HRZ/10HR

1. TB and HIV treatment can be started on the same day in children? (Please circle one answer.) 1. Yes
   1. No
   2. Not sure

1. Children with TB meningitis must always be given steroids? (Please circle one answer.)
   1. Yes
   2. No
   3. Not sure

# Prevention of TB

1. Which of the following children are eligible for TB preventive therapy? (Please circle all that are mentioned.)
   1. All children living with HIV irrespective of age
   2. Children living with HIV above 1 year
   3. All children
   4. Children living with HIV less than 1 year if in contact to a TB case
   5. Under 5 contacts to bacteriologically confirmed TB cases

1. What are the recommended regimens for TB preventive therapy in Zambia? (Please circle all that apply.)
   1. Ethambutol for 6 months
   2. Isoniazid for 3 months
   3. Isoniazid for 6 months
   4. Rifampicin and Isoniazid daily for 3 months
   5. Pyrazinamide for 3 months
   6. Rifapentin and isoniazid once a week for 3 months in children more than 2 years

1. Children with symptoms of TB can be started on TB preventive therapy (Please circle one response.) 1. Yes
   1. No
   2. Not sure

1. Before starting TB preventive therapy, the following **must** be done? (Please circle all that apply.)
   1. Gene Xpert
   2. Chest x-ray
   3. Symptom screening
   4. Mantoux

# TB attitudes

1. What do you feel is your role in diagnosis children with TB? (Cicle all that apply)
   1. Refer children with presumptive TB to the TB corner
   2. Request children with presumptive TB to submit sputum
   3. Fast track the children so that they can see a clinican quickly
   4. Document these children in the presumptive TB register
   5. I don’t know

1. Which statement is closest to your feeling about children with TB disease? (circle one answer.)
   1. “I feel compassion and desire to help.”
   2. “I feel compassion but I tend to stay away from these people.”
   3. “It is their problem and I cannot get TB.”
   4. “I fear them because they may infect me.”
   5. “I have no particular feeling.”
   6. Other (please explain):

1. Would you like to be more involved in TB activities?
   1. Yes
   2. No
   3. Not sure
2. What would be your reaction if you were asked to work at the TB corner? (circle one answer.) 1. I would refuse
   1. I don’t mind
   2. Scared
   3. Angry
   4. Happy
   5. Other

1. What is your biggest fear/concern about TB preventive therapy? (circle one answer.)
   1. Pill burden
   2. TB preventive therapy promotes drug resistant TB
   3. It does not have much benefit in a high burden setting
   4. Side effects
   5. Other

1. Do you think the benefits of TB preventive therapy outweigh the risks?
   1. Yes
   2. No
   3. Not sure

# Practices

1. Has your department provided any health education on childhood TB diagnosis or prevention in the past 1 week? (Circle one.)
   1. Yes
   2. No
   3. Not sure

1. How often do you interact with children with symptoms suggestive of TB? (Circle one.)
   1. Everyday
   2. More than once a week
   3. At least once a week
   4. Once a month
   5. Never

1. What do you do when you identify a child with symptoms of TB? (Circle all that apply.)
   1. Ask the patient to go to TB corner to submit a sputum sample
   2. Request the patient to submit a sputum sample
   3. Fast track the child
   4. Provide education on cough etiquette
   5. Document the child in the presumptive TB register
   6. Nothing

1. Which test do you often rely on to diagnose TB in children? (Circle one.)
   1. Sputum microscopy
   2. Gene Xpert
   3. Chest X-ray
   4. LAM
   5. Culture and DST
   6. Other
   7. N/A

1. What do you do for children not able to produce sputum? (Circle all that apply.)
   1. Prescribe antibiotics for 1 week and then ask them to return to the health facility for review
   2. Collect gastric aspirates
   3. Use chest x-ray to make a diagnosis
   4. Give mother sputum bottle to continue trying to get sputum from the child
   5. Request for LAM
   6. Other
   7. N/A

# TB awareness and sources of information

1. Do you feel well informed about TB?
   1. Yes
   2. No

1. Do you wish you could get more information about TB?
   1. Yes
   2. No

1. What do you think is the most effective way of improving knowledge, attitudes and practices towards childhood among health workers? (Please choose the three most effective sources.) 1. Trainings
   1. Orientation
   2. National guidelines
   3. CMEs
   4. Job aids
   5. Brochures, posters and other printed materials
   6. Other

1. What worries you the most when you think about TB?

Thank you very much for participating in our survey.
