# Supplementary material for: Knowledge, attitudes, and practices towards childhood tuberculosis among healthcare workers at two primary health facilities in Lusaka, Zambia
Source: PLoS One. 2024 Mar 11;19(3):e0287876. doi: 10.1371/journal.pone.0287876 (PMC10927107; doi:10.1371/journal.pone.0287876)
Supplement: Attachment — Submitted filename: Commrnts for PlOS ONE.docx [file pone.0287876.s003.docx]

The current study assesses the knowledge attitude and practices of HCWs regarding childhood TB. The topic is relevant and of global public health importance. However, I have the following comments and suggestions

1. **General comments:**

- Change health care to one word, healthcare on the study title and entire manuscript.
- Too many grammatical errors in the manuscript with poor punctuation: Full stops are included before the end of the reference in the majority of the sentences in the introduction section. Capital letter in the course of a sentence, lines 119, 218, 220, 266, 284, 351
- The authors should improve the quality of the manuscript, especially on the methodology by reading recently published papers on childhood TB KAP studies between 2021 and 202

https://pubmed.ncbi.nlm.nih.gov/36569997/

<https://pubmed.ncbi.nlm.nih.gov/35361143/>

<https://pubmed.ncbi.nlm.nih.gov/35197164/>

- The objectives of the study are not clear and the results are not coherent, not objectively presented within the text.

2. **Abstract**

- Abstract too long, more than 350 words, kindly review.
- Line 24 and 24: As per the WHO 2022 annual report, page 42, Zambia joined the list of 30 high MDR/RR-TB burden countries in 2022. So the sentence should read as Zambia is among the list of 30 high-burden countries for TB, HIV-associated TB, and MDR/RR-TB with a significant…
- Line 31: Add childhood before TB
- Line 32: Please indicate how the HCWs and or health facilities were sampled. Random sampling of the population is mentioned only under the strengths of the study
- Line 33: The phrase “the questionnaire data collected was later transcribed to an electronic system called DHIS 2” is not clear and confusing. Please kindly state how the data was analysis including the level of significance
- Line 34 and 45: The results are too long and do not present any finding on the study objective which is to understand the factors influencing the knowledge, 28 attitudes, and practices of health care workers (HCWs) regarding childhood TB in Zambia. Please kindly review. In addition, the conclusion is too long and does not correlate with the study findings.
- Line 56: Please remove survey as a keyword, not mentioned in the abstract or title of the manuscript.

Introduction

Line 61: The sentence “Majority (about 96%) of children who died from TB were not on treatment 62 (2)” is data from 2017 and does not correlate with the previous statement on globally…. Also, the sentence needs to be updated with data from the WHO 2022 TB annual report.

Line 63: The phrase should be made to children… is not clear and should be changed to should be among children

Line: 69: Pediatric TB diagnosis can be difficult. (4-6) should be corrected by including a full stop instead at the end of the sentence

Line 78, 80: Full stop at the end of the reference

Line 83: The objective mentioned is different from that at the level of the abstract. Please could you clarify on this?

Methods

- The methodology has a lot of missing information. Sampling of facility and HCWs, sample size, variables of interest, and not coherent
- Measurements: Your methodology does not explain how knowledge level was classified as bad, good, and unknown
- I recommend the authors should follow the STROBE guidelines

Line 87: What were the eligibility criteria for the HCWs to participate in the study? How were these HCWs sampled? The authors mentioned that registered healthcare workers (nurses, clinical officers, and medical officers) working at the health facility participated in the study. How sure are you that HCWs working in service units such as surgery, dentistry, and diabetic units for more than two years could provide correct information on childhood TB knowledge attitude, and practice?. I do not concur with your selection criteria.

Line 98: Please reference the different sources used to adopt the questionnaire. How did you ensure the validity of the questionnaire? Was it pre-tested? What about the different internal constructs of the questionnaire? Was Cronbach's alpha coefficient used to measure the internal consistency, or reliability, of the survey items?

Results:

Line 161: The different types of departments should be written in full to ease understanding or you indicated the full meaning at the end of the table

All the tables should be formatted in the standard way with three horizontal lines. See the example below for table 1

| **Table 1: Sociodemographic characteristics of the study population** | | |
| --- | --- | --- |
| Variable | Frequency (N) | Percentage (%) |
| **Age Group** |  |  |
| Under 30  41-50 |  |  |
| Over 50 |  |  |
| Unknown |  |  |
| **Sex** |  |  |
| Male | 66 | 27.7 |
| Female | 172 | 72.3 |
| **Department** |  |  |
| Outpatient Department |  |  |
| XXX |  |  |
| XXX |  |  |
| XXX |  |  |
| XXX |  |  |
| **Duration employed**  **Training** |  |  |

The variable “How long ago were you trained?” should be changed to training duration and the five modalities should be reduced to at most three (eg less than six months, Six months to two years, more than two years) in order to ease understanding and appreciate the data

Line 163: Include a heading on HCW knowledge

Line 202: sentence not clear please correct

210: I don’t think we say close association in scientific writing.

Line 214-216: The sentence “The characteristics and scores of healthcare workers in each category of the Knowledge, Attitudes, and Practices (KAP) survey are presented in Table 6: HCWs characteristics and 216 knowledge score below and in Table 5:” is not clear, please correct

**Discussion**

The discussion section is poorly written as references to similar studies are lacking. There is evidence of a poor literature review done on the paper.

**References**

The number of references (20) for this manuscript shows much literature was not done whereas many studies have been published on this topic between 21 and 22.

Line 374, 378, and 425: References are incomplete, kindly review

Line 67: Add a full stop at the end of the sentence
