# Supplementary material for: Knowledge, attitudes, and practices towards childhood tuberculosis among healthcare workers at two primary health facilities in Lusaka, Zambia
Source: PLoS One. 2024 Mar 11;19(3):e0287876. doi: 10.1371/journal.pone.0287876 (PMC10927107; doi:10.1371/journal.pone.0287876)
Supplement: Attachment — Submitted filename: Response to reviewers (2).docx [file pone.0287876.s004.docx]

First and foremost, we would like to thank the editor and the reviewers for taking time to review the submitted manuscript. We value the insightful comments provided, as addressing them has strengthened the quality of the manuscript. Our point-by-point responses to the comments are detailed in **bold** below.

**EDITOR’S COMMENTS**

1. Please ensure that your manuscript meets PLOS ONE's style requirements, including those for file naming. The PLOS ONE style templates can be found at

<https://journals.plos.org/plosone/s/file?id=wjVg/PLOSOne_formatting_sample_main_body.pdf>

and

<https://journals.plos.org/plosone/s/file?id=ba62/PLOSOne_formatting_sample_title_authors_affiliations.pdf>

**RESPONSE: The manuscript has been formatted to follow this guideline.**

2. Thank you for stating the following in your Competing Interests section:

“None Declared”

Please complete your Competing Interests on the online submission form to state any Competing Interests. If you have no competing interests, please state "The authors have declared that no competing interests exist.", as detailed online in our guide for authors at http://journals.plos.org/plosone/s/submit-now

This information should be included in your cover letter; we will change the online submission form on your behalf.

**RESPONSE: Thanks. This will be done when resubmitting the manuscript.**

3. In your Data Availability statement, you have not specified where the minimal data set underlying the results described in your manuscript can be found. PLOS defines a study's minimal data set as the underlying data used to reach the conclusions drawn in the manuscript and any additional data required to replicate the reported study findings in their entirety. All PLOS journals require that the minimal data set be made fully available. For more information about our data policy, please see http://journals.plos.org/plosone/s/data-availability.

Upon re-submitting your revised manuscript, please upload your study’s minimal underlying data set as either Supporting Information files or to a stable, public repository and include the relevant URLs, DOIs, or accession numbers within your revised cover letter. For a list of acceptable repositories, please see http://journals.plos.org/plosone/s/data-availability#loc-recommended-repositories. Any potentially identifying patient information must be fully anonymized.

Important: If there are ethical or legal restrictions to sharing your data publicly, please explain these restrictions in detail. Please see our guidelines for more information on what we consider unacceptable restrictions to publicly sharing data: http://journals.plos.org/plosone/s/data-availability#loc-unacceptable-data-access-restrictions. Note that it is not acceptable for the authors to be the sole named individuals responsible for ensuring data access.

We will update your Data Availability statement to reflect the information you provide in your cover letter.

**RESPONSE: The data will be made available as part of this publication.**

4. Please amend either the abstract on the online submission form (via Edit Submission) or the abstract in the manuscript so that they are identical.

**RESPONSE: This has been done, thank you.**

5. Your ethics statement should only appear in the Methods section of your manuscript. If your ethics statement is written in any section besides the Methods, please delete it from any other section.

**RESPONSE: It was in the methods section, thank you**

**REVIEWER 1**

1. Line 103- Knowledge of participant- Is this on TB or only on childhood TB? Please specify based on your KAP questionnaire

**RESPONSE: Thank you for the comment. This section has been restructured with that particular text removed. However, we have clarified that this was in relation to childhood TB.**

2. Line 111- Please write full form of CIDRZ if you have not mentioned prior.

**RESPONSE: The sentence has been revised, with the removal of the term CIDRZ.**

3. Please check line 201 and 202. The sentence is not aligned properly.

**RESPONSE: The sentence has been removed from the updated results section.**

4. Line 265-66- Among the variables, the significant association was found between KAP scores and Sex, Department, Trained- Please re-write this sentence to make it clearer.

**RESPONSE: This has been resolved with the updated results section.**

5. Line 291-92- "Staff from OPD, ART, and MCH had less understanding of TB infection control than those who did"- This sentence is not clear. What do you mean by "who did" in this sentence.

**RESPONSE: This has been resolved with the removal of the sentence in the updated results section.**

6. Better to present all responses on KAP questionnaire in the form of table in supplementary data section.

**RESPONSE: Thanks for the guide, all responses have been presented in the form of a table.**

7. Its better if you also mention the number of questions (out of 37) on Knowledge, Attitudes and Practices were there in KAP questionnaire.

**RESPONSE: This has been done, please see line 109-111.**

8. In statistical analysis part, you have only explained how you calculated Knowledge score but you have not mentioned about the scores for Attitudes and Practices. Please explain.

**RESPONSE: This has been addressed. Please see lines 124-131.**

9. Which data analysis software did you use for the analysis of KAP data? Please do mention.

**RESPONSE: STATA Statistical Software (Stata Corporation Version 14. College Station, Texas 77845, USA) was used. This has been included on line 120-121.**

10. You have mentioned "Passed" and "Failed" in Result section but what is your criteria for this classification. You should mention this in methods section.

**RESPONSE: The criteria has been added to the methods section. Please see line 128-131.**

11. I can see questions on Knowledge and practices in table 2 and 3 respectively but I cant see questions on Attitudes. Can you also present some questions on Attitudes.

**RESPONSE: This has been addressed in the revised result section. Please see line 188.**

12. In table 4 there is high non-response (more than 60%) rate for the question on TB infection control. What might be the reason for this? Was there high non-response rate for other questions as well?

**RESPONSE: We did not have a high non-response rate. In fact, overall, missing results are less than 5%. We have changed the presentation of the results to improve the clarity. Also, we have attached the questionnaire to provide insights into the nature of the questions.**

13. Heading of table 6 is not in line with data you presented in table as you have also presented Attitudes and Practices scores in table.

**RESPONSE: This has been addressed.**

**Discussion**

14. 8. Can you please include some references in discussion section. Some paragraphs in discussion sections are without any references. Its better to have references to support your findings in discussion section.

**RESPONSE: This has been addressed, thanks**

**REVIEWER 2**

1. Change health care to one word, healthcare on the study title and entire manuscript.

**RESPONSE: Thank you. This change has been made on the title and the rest of the document.**

2. Too many grammatical errors in the manuscript with poor punctuation: Full stops are included before the end of the reference in the majority of the sentences in the introduction section. Capital letter in the course of a sentence, lines 119, 218, 220, 266, 284, 351

**RESPONSE: These have been addressed. Some sentences have been reworded altogether to improve the grammar.**

3. The authors should improve the quality of the manuscript, especially on the methodology by reading recently published papers on childhood TB KAP studies between 2021 and 202

<https://pubmed.ncbi.nlm.nih.gov/36569997/>

<https://pubmed.ncbi.nlm.nih.gov/35361143/>

<https://pubmed.ncbi.nlm.nih.gov/35197164/>

**RESPONSE: We appreciate these resources; they have been useful in improving our methodology and results sections.**

4. The objectives of the study are not clear and the results are not coherent, not objectively presented within the text.

**RESPONSE: We have revised the objectives to make them clear. The sentence on objectives currently reads as: “We undertook a study to assess healthcare workers’ (HCWs) knowledge, attitudes, and practices towards childhood TB and the factors associated with good knowledge, attitudes, and practices towards childhood TB.” Please see line 91-94. We have also revised our results to align more closely with these objectives. Please review the results section for the relevant updates.**

5. Abstract too long, more than 350 words, kindly review.

**RESPONSE: The abstract has been revised to less than 350 words.**

6. Line 24 and 24: As per the WHO 2022 annual report, page 42, Zambia joined the list of 30 high MDR/RR-TB burden countries in 2022. So the sentence should read as Zambia is among the list of 30 high-burden countries for TB, HIV-associated TB, and MDR/RR-TB with a significant…

**RESPONSE: Thank you, we have revised the sentence as recommended. Please see line 24- 26.**

7. Line 31: Add childhood before TB.

**RESPONSE: Thank you, the sentence to which this comment corresponds has been removed.**

8. Line 32: Please indicate how the HCWs and or health facilities were sampled. Random sampling of the population is mentioned only under the strengths of the study.

**RESPONSE: Thank you. We have added that healthcare workers were selected through stratified random sampling. Please see line 32-33 (abstract section) as well as line 95 (methodology section).**

9. Line 33: The phrase “the questionnaire data collected was later transcribed to an electronic system called DHIS 2” is not clear and confusing. Please kindly state how the data was analysis including the level of significance.

**RESPONSE: Thank you, the sentence has been removed. Please see lines 31-37 on how data was analysed including the level of significance.**

10. Line 34 and 45: The results are too long and do not present any finding on the study objective which is to understand the factors influencing the knowledge, 28 attitudes, and practices of health care workers (HCWs) regarding childhood TB in Zambia. Please kindly review. In addition, the conclusion is too long and does not correlate with the study findings.

**RESPONSE: This is well noted. We have clarified the objective of the study in lines 27-29 and have restructured the results and conclusion to better align them to the study objectives.**

11. Line 56: Please remove survey as a keyword, not mentioned in the abstract or title of the manuscript.

**RESPONSE: This has been done.**

12. Line 61: The sentence “Majority (about 96%) of children who died from TB were not on treatment 62 (2)” is data from 2017 and does not correlate with the previous statement on globally…. Also, the sentence needs to be updated with data from the WHO 2022 TB annual report.

**RESPONSE: We have restructured this paragraph and used the most recent TB data. The revised paragraph reads, “Globally, an estimated 10.6 million people developed tuberculosis (TB) in 2022 of which about 12% were children. Of these children, only 46% were diagnosed and started on treatment. In Zambia, one of the 30 high-burden countries for Tuberculosis (TB), Human Immunodeficiency Virus (HIV)-associated TB, and multi-drug resistant/rifampicin resistant TB, only 68% of the estimated children with TB were diagnosed and started on treatment in 2022. Between 2020 and 2021, this proportion did not exceed 50% in either year.” Please see lines 62-67.**

13. Line 63: The phrase should be made to children… is not clear and should be changed to should be among children
    **RESPONSE: This is noted. The sentence has since been removed.**
14. Line: 69: Pediatric TB diagnosis can be difficult. (4-6) should be corrected by including a full stop instead at the end of the sentence

**RESPONSE: This was done. In addition, the correction was done to all other parts of the document that had this error.**

15. Line 78, 80: Full stop at the end of the reference.

**RESPONSE: Like the issue on number 14, this has addressed.**

16. Line 83: The objective mentioned is different from that at the level of the abstract. Please could you clarify on this?

**RESPONSE: This has addressed, the objectives have been aligned.**

17. The methodology has a lot of missing information. Sampling of facility and HCWs, sample size, variables of interest, and not coherent

**RESPONSE: This has addressed. More information has been added to the methodology section according to the STROBE guidelines and the requirements of the study.**

18. Measurements: Your methodology does not explain how knowledge level was classified as bad, good, and unknown

**RESPONSE: We have revised our methodology to address this concern. First, we added a sub-section on validation of correct responses. See line 111-113. We then explained how knowledge level was classified as good or poor, see line 121-131.**

19. I recommend the authors should follow the STROBE guidelines.

**RESPONSE: We appreciate your suggestion to adhere to the strobe guidelines. Following your recommendation, the necessary actions have been taken. Thank you.**

20. Line 87: What were the eligibility criteria for the HCWs to participate in the study? How were these HCWs sampled? The authors mentioned that registered healthcare workers (nurses, clinical officers, and medical officers) working at the health facility participated in the study. How sure are you that HCWs working in service units such as surgery, dentistry, and diabetic units for more than two years could provide correct information on childhood TB knowledge attitude, and practice?. I do not concur with your selection criteria.

**RESPONSE: We have clarified this- The selection criteria have been expanded on in the methodology. Basically, this involved all HCWs at departments that routinely provide TB screening and/or treatment of TB. Please see line 96-99.**

21. Line 98: Please reference the different sources used to adopt the questionnaire. How did you ensure the validity of the questionnaire? Was it pre-tested? What about the different internal constructs of the questionnaire? Was Cronbach's alpha coefficient used to measure the internal consistency, or reliability, of the survey items?

**RESPONSE: The questions were adapted primarily from the national pre-training test on childhood TB and the World Health Organisation (WHO) guide to developing KAP surveys. This has been added to line 108-109. We did not check the validated and reliability of the questionnaire. This has been included as a limitation. Please check line 276-278.**

22. All the tables should be formatted in the standard way with three horizontal lines. See the example below for table 1.

**RESPONSE: The tables have been edited according to the guidelines provided.**

23. The variable “How long ago were you trained?” should be changed to training duration and the five modalities should be reduced to at most three (eg less than six months, Six months to two years, more than two years) in order to ease understanding and appreciate the data

**RESPONSE: We have reworded “How long ago were you trained? to “Duration since previous training”. We have also reduced the responses to three. We have also applied this change to duration of working at the facility. Please see table 1 on line 154.**

24. Line 163: Include a heading on HCW knowledge.

**RESPONSE: This has been done.**

25. Line 202: sentence not clear please correct.

**RESPONSE: The sentence was revised to make it clearer.**

26. 210: I don’t think we say close association in scientific writing.

**RESPONSE: The results section was revised as appropriate.**

27. Line 214-216: The sentence “The characteristics and scores of healthcare workers in each category of the Knowledge, Attitudes, and Practices (KAP) survey are presented in Table 6: HCWs characteristics and 216 knowledge score below and in Table 5:” is not clear, please correct.

**RESPONSE: This entire results section has been changed, with the removal of the sentence.**

28. The discussion section is poorly written as references to similar studies are lacking. There is evidence of a poor literature review done on the paper.

**RESPONSE: The discussion section was revised.**

29. The number of references (20) for this manuscript shows much literature was not done whereas many studies have been published on this topic between 21 and 22.

**RESPONSE: We have included done a more thorough literature review and have more references.**

30. Line 374, 378, and 425: References are incomplete, kindly review.

**RESPONSE: This was resolved.**

31. Line 67: Add a full stop at the end of the sentence

**RESPONSE: This was resolved.**
